# Target weight achievement and ultrafiltration rate thresholds: potential patient implications

**DOI:** 10.1186/s12882-017-0595-5

**Published:** 2017-06-02

**Authors:** Jennifer E. Flythe, Magdalene M. Assimon, Robert A. Overman

**Affiliations:** 1 Division of Nephrology and Hypertension, Department of Medicine, University of North Carolina Kidney Center, UNC School of Medicine, 7024 Burnett-Womack CB #7155, Chapel Hill, NC 27599-7155 USA; 2 Cecil G. Sheps Center for Health Services Research, University of North Carolina, Chapel Hill, NC USA; 3 Department of Epidemiology, UNC Gillings School of Global Public Health, Chapel Hill, NC USA; 4 Division of Pharmaceutical Outcomes and Policy, UNC Eshelman School of Pharmacy, University of North Carolina, Chapel Hill, NC USA

**Keywords:** Hemodialysis, Target weight, Volume overload, Ultrafiltration rate, Quality Incentive program

## Abstract

**Background:**

Higher ultrafiltration (UF) rates and extracellular hypo- and hypervolemia are associated with adverse outcomes among maintenance hemodialysis patients. The Centers for Medicare and Medicaid Services recently considered UF rate and target weight achievement measures for ESRD Quality Incentive Program inclusion. The dual measures were intended to promote balance between too aggressive and too conservative fluid removal. The National Quality Forum endorsed the UF rate measure but not the target weight measure. We examined the proposed target weight measure and quantified weight gains if UF rate thresholds were applied without treatment time (TT) extension or interdialytic weight gain (IDWG) reduction.

**Methods:**

Data were taken from the 2012 database of a large dialysis organization. Analyses considered 152,196 United States hemodialysis patients. We described monthly patient and dialysis facility target weight achievement patterns and examined differences in patient characteristics across target weight achievement status and differences in facilities across target weight measure scores. We computed the cumulative, theoretical 1-month fluid-related weight gain that would occur if UF rates were capped at 13 mL/h/kg without concurrent TT extension or IDWG reduction.

**Results:**

Target weight achievement patterns were stable over the year. Patients who did not achieve target weight (post-dialysis weight ≥ 1 kg above or below target weight) tended to be younger, black and dialyze via catheter, and had shorter dialysis vintage, greater body weight, higher UF rate and more missed treatments compared with patients who achieved target weight. Facilities had, on average, 27.1 ± 9.7% of patients with average post-dialysis weight ≥ 1 kg above or below the prescribed target weight. In adjusted analyses, facilities located in the midwest and south and facilities with higher proportions of black and Hispanic patients and higher proportions of patients with shorter TTs were more likely to have unfavorable facility target weight measure scores. Without TT extension or IDWG reduction, UF rate threshold (13 mL/h/kg) implementation led to an average theoretical 1-month, fluid-related weight gain of 1.4 ± 3.0 kg.

**Conclusions:**

Target weight achievement patterns vary across clinical subgroups. Implementation of a maximum UF rate threshold without adequate attention to extracellular volume status may lead to fluid-related weight gain.

**Electronic supplementary material:**

The online version of this article (doi:10.1186/s12882-017-0595-5) contains supplementary material, which is available to authorized users.

## Background

Adequate fluid management is increasingly accepted as an important component of hemodialysis treatment adequacy. Existing data suggest that rapid fluid removal, extracellular volume expansion and large interdialytic weight gain (IDWG) are risk factors for morbidity and mortality among maintenance hemodialysis patients [1–6]. Growing interest in evaluating the quality of dialysis facility fluid management practices led to the recent consideration of two fluid-related clinical quality measures, an ultrafiltration (UF) rate measure and a target weight measure, for inclusion in the United States (U.S.) Centers for Medicare and Medicaid Services (CMS) End-Stage Renal Disease (ESRD) Quality Incentive Program (QIP) [7].

The ESRD QIP is the first mandatory, federal pay-for-performance program in the U.S. The program links Medicare payment for dialysis services to dialysis facility performance on quality measures. The current QIP includes quality measures on dialysis adequacy, anemia, mineral metabolism, and vascular access, among others. Measure development relies on Technical Expert Panels, public comment and measure endorsement by the National Quality Forum (NQF). Measures may be developed by CMS as well as by other stakeholder groups [8]. The UF rate and target weight measures were proposed as companion measures by a dialysis stakeholder coalition. The dual measures were intended to promote balance between too aggressive fluid removal and too conservative fluid removal [9]. In 2015, the NQF endorsed only the UF rate measure (now slated for QIP inclusion in payment year 2020), declining to recommend the target weight measure based on inadequate evidence and other concerns [7].

To reduce UF rates, providers can extend treatment times (TTs) or lower UF volumes. Patient preferences and facility operational burden may limit TT extension [10, 11]. To reduce UF volume without consequent volume expansion, IDWGs must be reduced. However, minimizing IDWG is difficult. If UF rate thresholds are adopted without adequate attention to extracellular volume status, patients may not achieve target weight and become volume-expanded over time. Mismatch between post-dialysis and target weights may occur for numerous reasons. Inaccurate target weight estimation may result in post-dialysis weights either above or below target weight. Intradialytic UF reduction or cessation with or without fluid bolus administration may lead to post-dialysis weights above target weights. Difficulties with target weight achievement should prompt clinical providers to reconsider either their approach to fluid removal or the target weight prescription. However, target weight adjustment is often overlooked. While target weight estimation is imperfect, target weight achievement has been associated with improved clinical outcomes [12, 13]. Target weight achievement is thus a plausible indicator of facility extracellular fluid management practices and a potential counterbalance for the UF rate quality measure.

We undertook this study to assess the proposed target weight quality measure by describing target weight achievement patterns across individuals and comparing dialysis facilities with higher (vs. lower) percentages of patients with failed target weight achievement. Also, we examine the associations of facility characteristics with higher (vs. lower) percentages of patients with failed target weight achievement, and calculate the theoretical amount of fluid-related weight that would be gained if an UF rate threshold of 13 mL/h/kg was implemented without concurrent IDWG reduction or TT extension.

## Methods

### Target weight achievement measure

We evaluated the Kidney Care Quality Alliance (KCQA)-proposed post-dialysis weight above or below target weight measure (Measure 2702) submitted to the May 2015 NQF. The measure was submitted as a companion measure to the KCQA-proposed UF rate measure (Measure 2701) [7]. The KCQA is a measure development committee sponsored by Kidney Care Partners, an alliance of dialysis stakeholders including patients, professionals, providers and manufacturers that aims to optimize kidney disease care through legislative, regulatory and quality activities [14].

Using 2012 data from a single dialysis organization, we analyzed the post-dialysis weight above or below target weight measure (henceforth referred to as the “target weight measure”) according to KCQA measure specification at the patient and facility levels (Table 1; Additional file 1: Table S1) [15]. The target weight measure reflects the percentage of patients at a dialysis facility with average post-dialysis weights ≥1 kg above or below prescribed target weight. The ±1 kg target weight difference was the difference magnitude proposed by measure developers. The measure is reported as a proportion (percentage of facility patients with average post-dialysis weights ≥1 kg above or below prescribed target weight). A lower score is better.

**Table 1:** Kidney Care Quality Alliance (KCQA) target weight measure specifications and selection criteria^a^

	Target Weight Measure
Description	% of patients in the facility with an average post-dialysis weight ≥ 1 kg above or below the prescribed target weight
Time window	12 months
Calculation period	Week of the monthly Kt/V
Numerator^a^	Number of patients in the facility with an average post-dialysis weight ≥ 1 kg above or below the prescribed target weight during the calculation period
Denominator	Total number of adult in-center HD patients at the reporting facility meeting inclusion criteria
Exclusions	1) PD or home HD patients; 2) Age < 18 years; 3) Treatment without post-weight and target weight; 4) ﻿﻿Patient present at reporting facility < 30 days; 5) Patient at facility with < 11 patients during month; 6) < 7 HD treatments at the facility in the reporting month
Algorithm	Calculated on a monthly basis and then averaged over year: 1) Sum denominator patients for each facility month2) Sum numerator patients for each facility month 3) Calculate monthly score = numerator patients / denominator patients for each month4) Calculate annual score = (sum of monthly scores) / # of months in reporting year
Score type	Rate / proportion
Score interpretation	Lower score more favorable

^a^The monthly target weight measure was calculated by dividing the total number of facility patients with an average post-dialysis weight ≥ 1 kg above or below target weight by the total number of facility hemodialysis patients meeting measure selection criteria on a monthly basis. For patients meeting the denominator selection criteria in the reporting month, the difference between the post-dialysis weight and the prescribed target weight for each treatment in the calculation period (during the week of the Kt/V assessment) was calculated. An average post-dialysis and prescribed target weight difference was then calculated from the treatments during the Kt/V assessment week. The number of patients in each facility with an average post-weight and target weight difference that was ± ≥ 1 kg during the calculation period were considered in the measure numerator. The annual target weight measure score was calculated by averaging facility monthly scores

*Abbreviations*: *HD* Hemodialysis, *PD* Peritoneal dialysis

The target weight measure was calculated by dividing the total number of facility patients with an average post-dialysis weight ≥ 1 kg above or below target weight by the total number of facility patients meeting measure selection criteria on a monthly basis. Per measure developer specifications, the monthly measure was computed as a mean of post-dialysis and target weight differences from treatments during the week of the monthly Kt/V assessment. Facility monthly measure percentages were averaged to create an annual facility measure score. Annual scores were dichotomized at the 75th percentile consistent with CMS facility performance measure evaluation.

Primary analyses considered the target weight measure as specified by KCQA. Secondary analyses considered a binary measure specification: patients with post-dialysis weight ≥ 1 kg *above* target weight were considered separately from patients with post-dialysis weight ≥ 1 kg *below* target weight. Additional secondary analyses considered facility target weight measure scores in conjunction with UF rate measure scores. The UF rate measure reflects the percentage of facility patients with average delivered UF rates ≥13 mL/h/kg and average delivered TTs <240 min. Measure specifics have been reported previously [11].

### Study design and selection criteria

This study was approved by the University of North Carolina at Chapel Hill Institutional Review Board. Data were taken from the records of 196,635 patients from 2449 U.S. facilities in the 2012 electronic medical record of a single dialysis provider. Cohort entry occurred between January 1 and November 30, 2012 and was based on rolling monthly application of selection criteria. Patients entered and exited the cohort throughout the year and could contribute to different facility monthly cohorts. This design mirrors real-world measure implementation and facilitated calculation of monthly and annual proportions of facility patients with post-dialysis weigh-arget weight differences outside of the proposed 1 kg threshold. Patient and facility eligibility criteria were assessed monthly and were based on the selection criteria set-forth by the measure developers. In-center hemodialysis patients aged ≥18 years who were present at the reporting facility for ≥30 days were included. Peritoneal dialysis and home hemodialysis patients, patients dialyzing at facilities with <11 patients, patients with incomplete post-dialysis weight or target weight data and patients with <7 treatments at the facility in the reporting month were excluded. These selection criteria were proposed by the measure developers to promote equitable assessment of target weight achievement across facilities and to focus on patients within facility control during the assessment period (i.e. to avoid undue influence from patients with prolonged hospitalizations or high percentages of missed outpatient treatments).

In analyses estimating theoretical fluid-related weight gain after application of an UF rate threshold of 13 mL/h/kg, we considered patients with complete treatment data from February 2012 (i.e. no missed outpatient treatments and no missing pre-weight, post-weight or target weight data) and who survived 60 days after the end of February 2012. In these analyses, IDWG was calculated as: pre-dialysis weight minus post-dialysis weight from prior treatment. Prescribed UF rate (mL/h/kg) was calculated as: IDWG (mL)/ prescribed TT (h)/ post-dialysis weight (kg).

### Data collection

Study data were taken from the dialysis provider’s electronic medical record. Demographic and co-morbid data were documented at the time of admission to the dialysis organization and updated based on clinical course. Clearance (Kt/V) was measured at least monthly per standard protocols. When more than one monthly Kt/V was available, the last monthly value was used. Dialysis variables including pre- and post-dialysis weights, target weight and TTs were recorded each treatment. Zip codes were used for facility geographic region assignment.

### Statistical analyses

Analyses were performed using SAS version 9.4 (SAS Institute, Cary, NC). Patient and facility characteristics were described as numbers (percentages) for categorical variables and means ± standard deviations or medians [quartile 1, quartile 3] for continuous variables. Target weight achievement (vs. not) was summarized across the full cohort. Differences in facility characteristics across annual target weight facility measure scores (≤75th percentile vs. >75th percentile) were assessed by Chi-square tests for categorical variables and Student’s T-test for continuous variables. Adjusted associations between facility characteristics and annual facility target weight measure scores were determined using multivariable logistic regression models. The outcome of interest was the facility annual target weight measure score (≤75th vs. >75th percentile). Two-tailed *P* values <0.05 indicated statistical significance.

To demonstrate potential theoretical fluid-related weight gain resulting from implementation of an UF rate threshold of 13 mL/h/kg without concurrent IDWG reduction or TT extension, we calculated fluid-related weight gains using observed IDWGs and prescribed TTs during consecutive treatments in a single month. For each treatment, we computed the maximum UF volume (L) allowable under the constraint of a maximum prescribed UF rate of 13 mL/h/kg using the patient’s prescribed TT and post-dialysis weight from the previous treatment. The theoretical per treatment weight change (kg) was calculated as: actual IDWG minus maximum allowable UF. Cumulative fluid-related weight change was then calculated by summing weight changes across the study month of interest. Detailed methods are provided in Additional file 1: Table S2.

## Results

### Cohort characteristics and target weight measure description

**Fig. 1:**
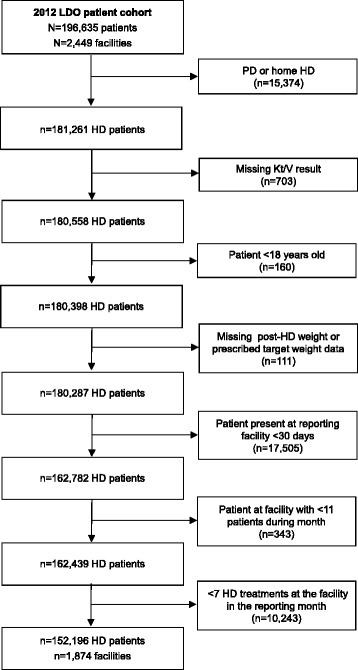
Flow diagram of the unique target weight measure cohort based on application of measure selection criteria on a rolling monthly basis. Abbreviations: LDO, large dialysis organization; HD, hemodialysis; PD, peritoneal dialysis

Target weight measure analyses considered 152,196 unique patients from 1874 facilities (Fig. 1). The mean age was 61 years, 36.4% were black, 17.3% were Hispanic, 24.9% had heart failure and 70.3% had prescribed TTs <240 min in the first reporting month of 2012. The cohort was similar to the broader U.S. hemodialysis population in terms of age, race, heart failure status and prescribed dialysis TT [16]. On average, target weight prescriptions were changed 0.55 ± 0.80 times per month.

The annual patient-level mean post-dialysis-target weight difference was 0.3 ± 1.2 kg. Monthly patient-level mean post-dialysis-target weight differences were stable over the year (Additional file 1: Table S3). Facilities had, on average, 27.1 ± 9.7% of patients with average post-dialysis weight ≥ 1 kg above or below the prescribed target weight. Monthly facility target weight measure scores varied over the year with scores peaking in winter (29.0 ± 11.9%) and nadiring in summer (25.7 ± 11.0%) (Additional file 1: Figure S1 and Table S3). Secondary analyses considering monthly post-dialysis-target weight differences specified as ≥1 kg *below* vs. ≥1 kg *above* target weight are displayed in Additional file 1: Table S4.

### Patient and facility characteristics across target weight achievement groups

Patients who did not achieve target weight (post-dialysis weight ≥ 1 kg above or below target weight) tended to be younger, black and dialyze via catheter, and had shorter dialysis vintage, greater body weight, higher UF rate and more missed treatments compared with patients who achieved target weight. When patients who missed target weight were dichotomized by above or below missed target weight status, patients with mean post-dialysis weights ≥1 kg *above* target weight were more likely to be younger, have histories of heart failure and diabetes, longer dialysis vintage, greater body weight, UF volume and UF rate and fewer missed treatments, compared to patients with mean post-dialysis weights ≥1 kg *below* target weight (Table 2).

**Table 2:** Patient characteristics by target weight achievement group as of the first reporting month in 2012^a^

Characteristic	Achieved target weight^b^ (*n* = 104,377)	Missed target weight^c^ (*n* = 47,819)	Below target weight^d^ (*n* = 17,466)	Above target weight^e^ (*n* = 30,353)
Post-dialysis weight and target weight difference				
Mean ± SD	0.0 ± 0.5;	0.6 ± 3.3;	−2.7 ± 2.4;	2.5 ± 1.9;
Median [Q1, Q3]	0.0 [−0.3, 0.4]	1.3 [−1.5, 2.2]	−1.9 [−3.2, −1.3]	1.9 [1.4, 2.9]
Age (years)	62.9 ± 15.0	61.0 ± 14.9	62.5 ± 15.0	60.2 ± 14.8
Female sex	46,842 (44.9)	21,604 (45.2)	8100 (46.4)	13,504 (44.5)
Black race	37,129 (35.6)	18,210 (38.1)	6399 (36.6)	11,811 (38.9)
Hispanic ethnicity	18,268 (17.5)	8079 (16.9)	3039 (17.4)	5040 (16.6)
Time on dialysis				
< 1 years	33,105 (31.7)	16,770 (35.1)	7904 (45.3)	8866 (29.2)
1–5 years	45,341 (43.4)	20,371 (42.6)	6216 (35.6)	14,155 (46.6)
> 5 years	25,931 (24.8)	10,678 (22.3)	3346 (19.2)	7332 (24.2)
Access type				
Graft	18,840 (18.1)	8222 (17.2)	2675 (15.4)	5547 (18.3)
Fistula	58,615 (56.2)	23,714 (49.7)	7417 (42.6)	16,297 (53.8)
Catheter	26,764 (25.7)	15,781 (33.1)	7330 (42.1)	8451 (27.9)
Pre-dialysis SBP				
< 100 mmHg	1202 (1.2)	832 (1.7)	347 (2.0)	485 (1.6)
100–129 mmHg	19,979 (19.1)	10,036 (21.0)	4000 (22.9)	6036 (19.9)
130–159 mmHg	50,801 (48.7)	21,782 (45.6)	8009 (45.9)	13,773 (45.4)
≥ 160 mmHg	32,388 (31.0)	15,163 (31.7)	5108 (29.2)	10,055 (33.1)
History of heart failure	25,204 (24.1)	12,679 (26.5)	3417 (19.6)	9262 (30.5)
History of diabetes	42,687 (40.9)	20,335 (42.5)	6144 (35.2)	14,191 (46.8)
Post-dialysis weight (kg)	78.7 ± 21.5	83.9 ± 25.0	80.0 ± 23.5	86.2 ± 25.6
Prescribed TT <240 min	74,970 (71.8)	31,982 (66.9)	12,157 (69.6)	19,825 (65.3)
UF volume (L)	2.4 ± 1.2	2.7 ± 1.7	2.3 ± 1.8	2.9 ± 1.5
Prescribed UF rate (mL/h/kg)	8.6 ± 4.5	9.3 ± 5.6	7.7 ± 6.0	10.2 ± 5.2
Missed treatments ≥3	11,307 (10.8)	7921 (16.6)	3686 (21.1)	4235 (14.0)
eKt/V	1.7 ± 0.3	1.6 ± 0.3	1.6 ± 0.3	1.6 ± 0.3

^a^Patient *N* = 152,196. Values presented as means ± SDs, medians [quartile 1, quartile 3] or numbers (percentages). Because patients entered and exited the cohort according to selection criteria application on a rolling monthly basis, the first reporting month varied across patients

^b^Average post-dialysis weight *< 1 kg above or below* the prescribed target weight

^c^Average post-dialysis weight *≥ 1 kg above or below* the prescribed target weight

^d^Average post-dialysis weight *≥ 1 kg below* the prescribed target weight (below depicted by a negative sign)

^e^Average post-dialysis weight *≥ 1 kg above* the prescribed target weight

Facilities in the highest measure quartile (>32.9% of patients with average post-dialysis weights ≥1 kg above or below the prescribed target weight), indicating worse performance, were more likely to be located in the western U.S., treat a greater number of patients, and have more black and Hispanic patients and more patients with TTs <240 min and UF rates ≥13 mL/h/kg (Table 3). Facilities in the highest measure quartile had significantly fewer target weight prescription changes per month compared to facilities in the lower target weight measure quartiles (0.39 ± 0.65 vs. 0.60 ± 0.84; *p* = 0.005). In secondary analyses, facilities falling in the highest target weight measure quartile *and* the highest UF rate measure quartile (>32.9% of patients with average post-dialysis weights ≥1 kg above or below the target weight and >20.3% of patients with average UF rates ≥13 mL/h/kg and average TTs <240 min) were more likely to be located in the western U.S., treat more patients and have more Hispanic patients (Table 4).

**Table 3:** Facility characteristics by annual facility target weight measure score^a^

	Target Weight Measure^b^		
Characteristic	≤75th measure percentile (*n* = 1406)	>75th measure percentile (*n* = 468)	*p* ^c^
Geographic region			
Northeast	139 (9.9)	56 (11.9)	≤0.01
Midwest	361 (25.7)	90 (19.2)	
South	659 (46.9)	195 (41.6)	
West	246 (17.5)	128 (27.3)	
Monthly facility size (number of patients)	59.7 ± 36.9	63.8 ± 40.0	<0.05
Household income ($)^d^	52,251 ± 20,018	50,489 ± 19,735	NS
Age < 50 years	18.2 ± 8.0	20.3 ± 7.9	<0.01
Black race	33.8 ± 29.7	40.6 ± 32.8	<0.01
Black race >50% of facility patients	412 (29.3)	182 (38.8)	<0.01
Hispanic ethnicity	13.0 ± 19.8	16.2 ± 22.8	<0.01
Hispanic ethnicity >25% of facility patients	241 (17.2)	111 (23.7)	<0.01
Heart failure balance >25% of facility patients	808 (57.5)	254 (54.2)	NS
Prescribed TT <240 min	70.9 ± 19.0	73.7 ± 17.6	<0.01
Prescribed TT <240 min >50% of facility patients	1190 (84.7)	422 (90.0)	<0.01
Prescribed UF rate ≥ 13 mL/h/kg	18.9 ± 10.1	20.3 ± 9.8	<0.01
Prescribed UF rate ≥ 13 mL/h/kg >33% of facility patients	121 (8.6)	51 (10.9)	NS

^a^Facility *N* = 1874. Values presented as numbers (percentages) of facilities or means ± SDs of the percentage of facility patients. Using prescribed TT as an example, a mean of 70.9% of patients at facilities in the ≤75th measure percentile had a prescribed TT <240 min as compared to a mean of 73.7% of patients at facilities in the >75th measure percentile

^b^The 75th percentile represents 32.9% of facility patients with post-dialysis weight ≥ 1 kg above or below the prescribed target weight. Measure 75th percentile reflects the 75th percentile of the annual measure score, and facility data reflect the January 2012 cohort data

^c^Significance was assessed by Chi-square test for categorical variables and Student’s T-test for continuous variables

^d^Mean of the median household incomes. Income data was obtained from 2010 U.S. Census data based upon dialysis facility zip code

**Table 4:** Facility characteristics by annual facility ultrafiltration rate and target weight measure scores^a^

	Ultrafiltration Rate Measure and Target Weight Measure^b^		
Characteristic	≤75th percentile for both measures (*n* = 1062)	>75th percentile for one but not both measures (*n* = 682)	>75th percentile for both measures (*n* = 130)
Geographic region			
Northeast	115 (10.8)	75 (11.0)	5 (3.8)
Midwest	267 (25.1)	157 (23.0)	27 (20.8)
South	521 (49.1)	286 (41.9)	47 (36.2)
West	159 (15.0)	164 (24.0)	51 (39.2)
Monthly facility size (number of patients)	58.6 ± 35.9	62.6 ± 39.5	68.2 ± 41.4
Household income ($)^c^	51,870 ± 20,062	52,300 ± 19,911	48,750 ± 19,211
Age < 50 years	18.1 ± 8.1	19.0 ± 8.0	21.5 ± 7.2
Black race	35.1 ± 30.4	36.3 ± 30.9	34.0 ± 31.4
Black race >50% of facility patients	337 (31.7)	219 (32.1)	38 (29.2)
Hispanic ethnicity	12.1 ± 19.1	14.8 ± 21.0	22.8 ± 26.8
Hispanic ethnicity >25% of facility patients	163 (15.3)	143 (21.0)	46 (35.4)
Heart failure balance >25% of facility patients	603 (56.8)	383 (56.2)	76 (58.5)
Prescribed TT <240 min	67.1 ± 19.6	76.5 ± 16.2	83.0 ± 10.7
Prescribed TT <240 min >50% of facility patients	849 (79.9)	633 (92.8)	130 (100.0)
Prescribed UF rate ≥ 13 mL/h/kg	15.4 ± 7.5	23.2 ± 10.6	30.4 ± 8.5
Prescribed UF rate ≥ 13 mL/h/kg >33% of facility patients	19 (1.8)	107 (15.7)	46 (35.4)

^a^Facility *N* = 1874. Values presented as numbers (percentages) of facilities or means ± SDs of the percentage of facility patients

^b^The 75th percentile of the target weight measure represents 32.9% of facility patients with post-dialysis weight ≥ 1 kg above or below the prescribed target weight. The 75th percentile of the ultrafiltration rate measure represents 20.3% of facility patients with UF rate ≥ 13 mL/h/kg and prescribed TT <240 min. Measure 75th percentile reflects the 75th percentile of the annual measure score, and facility data reflect the January 2012 cohort data

^c^Mean of the median household incomes. Income data was obtained from 2010 U.S. Census data based upon dialysis facility zip code

In adjusted analyses, dialysis facilities located in the midwest and south (vs. west), and facilities with higher proportions of black and Hispanic patients and higher proportions of individuals with TTs < 240 min were more likely to have unfavorable (i.e. higher) facility target weight measure scores (Table 5).

**Table 5:** Adjusted associations between facility characteristics and less favorable facility target weight measure score (>75th percentile)^a,b^

Characteristic	Adjusted OR (95% CI)
Geographic region	
West	1.00 (reference)
Midwest	2.33 (1.61–3.37)
South	2.68 (1.91–3.77)
Northeast	1.34 (0.88–2.02)
Monthly facility size (number of patients)	
≤ 25	1.93 (0.81–4.56)
26–50	1.86 (0.91–3.77)
51–100	1.51 (0.91–3.80)
≥ 101	1.00 (reference)
Household income > median zip code income ($)^c^	1.20 (0.96–1.50)
Age (per 10 years)	1.00 (1.00–1.01)
Black race >50% of facility patients	2.34 (1.78–3.07)
Hispanic ethnicity >25% of facility patients	1.45 (1.06–1.99)
Heart failure balance >25% of facility patients	0.79 (0.63–1.00)
Prescribed TT <240 min in >50% of facility patients	1.62 (1.15–2.28)
Prescribed UF rate ≥ 13 mL/h/kg in >33% of facility patients	1.31 (0.91–1.88)

^a^Facility *N* = 1874. The multivariable binary logistic regression model with a dependent outcome of annual target weight measure score ≤ 75th percentile vs. >75th percentile was adjusted for geographic region (midwest, south, northeast vs. west), unit size (≤25, 26–50, 51–100 vs. ≥101 patients), household income (≤ vs. > median zip code income), age (per 10 years), black race (≤50% vs. >50% of facility patients), Hispanic ethnicity (≤25% vs. >25% of facility patients), heart failure balance (≤25% vs. >25% of facility patients), prescribed TT <240 min (≤50% vs. >50% of facility patients), and prescribed UF rate ≥ 13 mL/h/kg (≤33% vs. >33% of facility patients)

^b^The 75th percentile represents 32.9% of facility patients with post-dialysis weight ≥ 1 kg above or below the prescribed target weight. Measure 75th percentile reflects the 75th percentile of the annual measure score, and facility data reflect the January 2012 cohort data

^c^ Mean of the median household incomes. Income data was obtained from 2010 U.S. Census data based upon dialysis facility zip code

### Individual fluid-related weight gains in the setting of Ultrafiltration rate thresholds

**Fig. 2:**
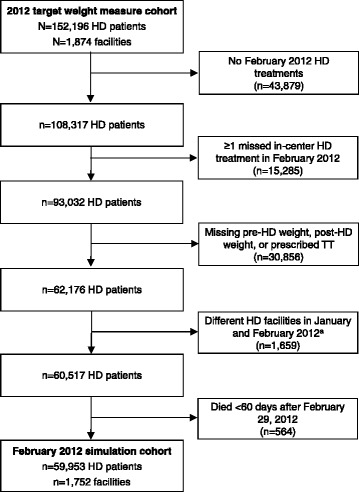
Flow diagram of the restricted cohort for fluid-related weight gain analyses. ^a^ To calculate the IDWG at the first February hemodialysis treatment, we required the post-dialysis weight from the last treatment in January and hence required patients to receive treatment at the same facility in January and February. Abbreviations: HD, hemodialysis; IDWG, interdialytic weight gain

**Fig. 3:**
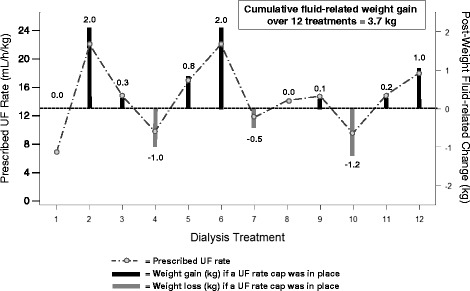
Illustration of fluid-related weight gain calculation for a single representative patient with varying observed IDWGs and UF rates over 12 consecutive treatments.The light gray circles and corresponding dashed lines represent the prescribed UF rates on the basis of observed IDWGs and prescribed TTs *without application* of a UF rate threshold across the study month. The horizontal dashed reference line represents a UF rate of 13 ml/h/kg. For treatments with prescribed UF rates ≥13 mL/h/kg, the black bars represent the calculated amount of fluid-related weight that would be gained if a UF rate threshold of 13 mL/h/kg was applied. For treatments with prescribed UF rates <13 mL/h/kg, the gray bars represent the calculated amount of fluid-related weight that would be lost (from prior fluid-related weight gains) if the UF rate was allowed to rise to a level of 13 mL/h/kg. Detailed methods and an example of the 1-month cumulative fluid-related weight gain computation are provided in Additional file 1: Table S2. Abbreviations: IDWG, interdialytic weight gain; TT, treatment time; UF, ultrafiltration

Among 59,953 patients in the fluid-related weight gain calculation cohort (Fig. 2), 11,161 (18.6%) had average post-dialysis weights ≥1 kg above or below the prescribed target weight in February 2012. Fig. 3 depicts a single patient’s 1-month weight gain in response to application of an UF rate threshold of 13 mL/h/kg with no change in observed IDWGs and prescribed TTs. Overall, the mean 30-day cumulative weight difference was 1.4 ± 3.0 kg. Patients with heart failure, shorter TT, greater IDWG and higher UF rates had greater cumulative weight gains (Table 6). When weight gains were considered at the facility level, on average, 17.4 ± 10.8% of facility patients had 30-day weight gains ≥2 kg, 13.3 ± 9.6% had 30-day weight gains ≥3 kg and 10.6 ± 8.5% had 30-day weight gains ≥4 kg. Higher facility percentages at each fluid-related weight gain level were observed among females, patients with heart failure, shorter TTs, greater IDWGs and higher UF rates (Table 7).

**Table 6:** Calculated patient-level fluid-related weight gains after institution of an UF rate threshold of 13 mL/h/kg (February 2012)^a^

	30-Day Cumulative Difference in Post-dialysis Weight (kg)		30-Day Cumulative % Difference in Post-dialysis Weight (%)	
Group	Mean ± SD	Median [Q1, Q3]	Mean ± SD	Median [Q1, Q3]
Overall	1.4 ± 3.0	0.1 [0.0, 1.2]	2.2 ± 5.0	0.2 [0.0, 1.7]
Females	1.4 ± 3.0	0.1 [0.0, 1.3]	2.5 ± 5.5	0.3 [0.0, 2.0]
Males	1.3 ± 2.9	0.1 [0.0, 1.1]	2.0 ± 4.5	0.2 [0.0, 1.6]
Black race	1.1 ± 2.5	0.0 [0.0, 0.9]	1.7 ± 3.9	0.1 [0.0, 1.4]
Non-black race	1.5 ± 3.2	0.1 [0.0, 1.3]	2.5 ± 5.4	0.3 [0.0, 2.0]
Hispanic ethnicity	1.8 ± 3.3	0.3 [0.0, 1.8]	2.9 ± 5.7	0.5 [0.0, 2.7]
Non-Hispanic ethnicity	1.3 ± 2.9	0.1 [0.0, 1.0]	2.0 ± 4.7	0.2 [0.0, 1.6]
No Heart Failure	1.3 ± 2.8	0.1 [0.0, 1.1]	2.0 ± 4.7	0.2 [0.0, 1.6]
Heart Failure	1.6 ± 3.3	0.1 [0.0, 1.4]	2.5 ± 5.4	0.3 [0.0, 2.0]
TT < 240 Minutes	1.6 ± 3.2	0.2 [0.0, 1.5]	2.6 ± 5.4	0.4 [0.0, 2.2]
TT ≥ 240 Minutes	0.8 ± 2.0	0.0 [0.0, 0.6]	1.1 ± 3.0	0.0 [0.0, 0.9]
IDWG <2 kg	0.4 ± 1.3	0.0 [0.0, 0.2]	0.8 ± 2.5	0.0 [0.0, 0.5]
IDWG ≥2 kg	1.8 ± 3.4	0.4 [0.0, 1.8]	2.8 ± 5.6	0.6 [0.0, 2.5]
IDWG <3 kg	0.7 ± 1.9	0.0 [0.0, 0.5]	1.3 ± 3.5	0.0 [0.0, 1.0]
IDWG ≥3 kg	2.4 ± 3.9	0.7 [0.0, 2.8]	3.6 ± 6.4	0.9 [0.0, 3.7]
IDWG <4 kg	1.0 ± 2.4	0.0 [0.0, 0.8]	1.7 ± 4.3	0.0 [0.0, 1.3]
IDWG ≥4 kg	3.1 ± 4.5	1.2 [0.2, 3.9]	4.4 ± 7.1	1.4 [0.3, 5.1]
UF rate < 13 mL/h/kg	0.5 ± 1.4	0.0 [0.0, 0.4]	0.8 ± 2.2	0.0 [0.0, 0.7]
UF rate ≥ 13 mL/h/kg	4.1 ± 4.6	2.3 [0.8, 6.0]	6.6 ± 7.9	3.4 [1.1, 9.5]

^a^Estimation of fluid-related weight gains were based on the February 2012 fluid-related weight gain calculation cohort (*N* = 59,953)

*Abbreviations*: *IDWG* Interdialytic weight gain, *TT* Treatment time, *UF* Ultrafiltration

**Table 7:** Percentage of facility patients with 30-day cumulative fluid-related weight gains above varied thresholds after institution of an UF rate threshold of 13 mL/h/kg overall and by subgroup (February 2012)^a^

Group	% of facility patients with a 30-day cumulative fluid-related weight gain		
	≥ + 2 kg	≥ + 3 kg	≥ + 4 kg
Overall	17.4 ± 10.8; 16.0 [9.5, 24.1]	13.3 ± 9.6; 11.6 [6.5, 18.8]	10.6 ± 8.5; 9.1 [4.4, 15.6]
Sex			
Female	18.6 ± 14.7; 16.7 [7.9, 27.3]	14.5 ± 13.4; 12.5 [3.3, 22.2]	11.5 ± 11.8; 9.5 [0.0, 18.2]
Male	16.5 ± 12.9; 14.3 [7.1, 24.0]	12.5 ± 11.5; 10.8 [3.4, 19.0]	9.9 ± 10.3; 7.7 [0.0, 15.4]
Race			
Black	15.1 ± 18.4; 11.1 [0.0, 22.5]	11.1 ± 16.5; 6.3 [0.0, 16.7]	8.7 ± 14.9; 3.2 [0.0, 12.5]
Non-black	19.4 ± 15.3; 17.1 [9.1, 27.3]	15.2 ± 13.8; 13.0 [5.3, 22.2]	12.2 ± 12.4; 10.0 [1.9, 18.2]
Ethnicity			
Hispanic	24.6 ± 28.7; 17.2 [0.0, 34.7]	19.8 ± 26.5; 11.6 [0.0, 28.6]	16.2 ± 24.4; 6.7 [0.0, 23.1]
Non-Hispanic	16.6 ± 11.2; 15.2 [8.8, 23.1]	12.5 ± 10.0; 10.7 [5.6, 18.2]	9.9 ± 9.0; 8.0 [3.4, 14.7]
Heart Failure			
(−) Heart failure	16.5 ± 11.9; 15.4 [7.7, 23.5]	12.5 ± 10.6; 10.7 [5.0, 18.2]	9.8 ± 9.6; 8.0 [1.8, 14.5]
(+) Heart failure	20.3 ± 20.3; 16.7 [0.0, 31.0]	15.9 ± 18.7; 11.1 [0.0, 25.0]	12.9 ± 17.1; 7.7 [0.0, 20.0]
Prescribed TT			
< 240 min	19.6 ± 12.8; 18.8 [10.3, 27.8]	15.1 ± 11.4; 13.3 [7.1, 22.1]	12.2 ± 10.2; 11.1 [4.5, 18.2]
≥ 240 min	10.7 ± 15.6; 5.7 [0.0, 16.7]	7.3 ± 13.3; 0.0 [0.0, 11.1]	5.2 ± 11.4; 0.0 [0.0, 6.7]
IDWG			
< 2 kg	6.0 ± 10.6; 0.0 [0.0, 10.0]	3.9 ± 8.3; 0.0 [0.0, 5.7]	2.8 ± 6.9; 0.0 [0.0, 0.0]
≥ 2 kg	22.3 ± 13.9; 21.1 [12.5, 30.8]	17.3 ± 12.6; 15.4 [8.3, 25.0]	13.8 ± 11.2; 12.0 [5.6, 20.0]
< 3 kg	10.1 ± 9.8; 8.1 [2.4, 15.0]	7.2 ± 8.1; 5.3 [0.0, 11.1]	5.5 ± 7.2; 3.4 [0.0, 8.3]
≥ 3 kg	28.7 ± 19.5; 26.9 [15.4, 40.0]	22.6 ± 18.2; 20.0 [10.0, 33.3]	18.3 ± 16.5; 16.7 [6.3, 26.9]
< 4 kg	13.5 ± 10.0; 12.0 [6.3, 19.0]	10.1 ± 8.7; 8.3 [3.7, 14.3]	7.9 ± 7.6; 6.3 [0.0, 11.8]
≥ 4 kg	36.7 ± 28.1; 33.3 [16.7, 50.0]	29.0 ± 27.0; 25.0 [0.0, 44.4]	23.8 ± 25.5; 20.0 [0.0, 35.7]
Prescribed UF rate			
< 13 mL/h/kg	7.3 ± 6.8; 6.3 [2.1, 10.5]	4.7 ± 5.6; 3.6 [0.0, 7.1]	3.3 ± 4.9; 0.0 [0.0, 5.1]
≥ 13 mL/h/kg	53.4 ± 26.7; 50.0 [35.7, 68.8]	43.8 ± 27.2; 42.9 [25.0, 59.4]	36.3 ± 26.7; 33.3 [18.2, 50.0]

^a^Estimation of weight gains were based on the February 2012 fluid-related weight gain calculation cohort (patient *N* = 59,953 and facility *N* = 1752). Values presented as means ± SDs; medians [quartile 1, quartile 3]

*Abbreviations*: *IDWG* Interdialytic weight gain, *TT* Treatment time, *UF* Ultrafiltration

## Discussion

In this study we examined target weight achievement patterns, the proposed but unendorsed target weight clinical performance measure and estimated the theoretical cumulative fluid-related weight gains that could result from UF rate threshold implementation. Patient-level target weight achievement patterns remained generally stable over the year, but facility target weight measure scores fluctuated, with the highest percentage of facility patients missing target weight in winter. Patients who did not achieve target weight were more likely to be younger, black, heavier, dialyze via catheter, have higher UF rates and more missed treatments compared to patients who achieved target weight. Facilities with higher percentages of patients who did not achieve target weight were more likely to be located in the western U.S., treat more patients and have more black and Hispanic patients compared with facilities with lower percentages. Without concurrent TT extension or IDWG reduction, implementation of an UF rate threshold of 13 mL/h/kg led to a mean theoretical fluid-related weight gain of 1.4 ± 3.0 kg with greater gains among females and patients with heart failure, shorter TTs and greater IDWGs.

Among maintenance hemodialysis patients, overly rapid fluid removal can lead to end-organ ischemic injury and associated morbidity [5, 17]. Alternatively, *insufficient* fluid removal can lead to volume overload and associated adverse outcomes [3, 4]. In practice, clinicians seek to balance the ischemic consequences of overly aggressive UF with the volume overload consequences of overly conservative UF. To lower UF rates, providers can increase TTs or lower UF volumes. In general, patients are averse to longer TTs [10], and a recent analysis demonstrated substantial facility operational burden (clinic resources, staff time, disruption of patient and transportation schedules) from extended treatments [11]. Furthermore, IDWGs remain high despite inclusion of salt and fluid restriction education in dietary programs [18]. These realities raise legitimate concern that UF rate reduction may be challenging in some settings. Thus, when extracellular volume status is not adequately emphasized, UF rate threshold implementation may lead to fluid-related weight gain. Target weight achievement assessment is one potential approach to balancing potential unintended consequences from UF rate limitation.

Clinical quality measures are tools to measure and track health care service quality. The Agency for Healthcare and Research Quality cites importance, scientific soundness and feasibility as desirable attributes. Measures should also have the capacity to be stratified by subgroups to evaluate for care disparities across populations [19]. Our data highlight differences among patients (and facilities) who achieve target weight (vs. not). We reported that, compared to patients who achieved target weight, patients who did not achieve target weight were younger, more likely to be black and had greater body weights, among other differences. Facilities with greater proportions of patients who did not achieve target weight were more likely be located in the western U.S., treat more patients and have more black and Hispanic patients compared with facilities with lower percentages. These differences suggest that facility case-mix adjustments to target weight measure scores may be needed.

The importance of an extracellular volume status measure as a counter-balance to an UF rate measure should not be underestimated, particularly in the U.S. where TTs are notably shorter than elsewhere in the world [20]. In previous analyses, we demonstrated that facilities treating >100 patients would require, on average, 33 additional treatment hours per week to lower UF rates to 13 mL/h/kg without concurrent IDWG reduction [11]. Such TT extension has implications for staffing, facility costs and, most importantly, other patients’ treatment schedules. If facilities are not able to meet these operational demands or if patients decline TT extension and IDWGs are not sufficiently lowered, fluid-related weight gain will occur in response to UF rate reduction. In this study we demonstrated that, on average, patients would gain 1.4 ± 3.0 kg under this paradigm. Weight gains were higher among non-blacks and patients with heart failure, and shorter treatment times. When considered at the facility level, on average, 17% of facility patients gained ≥2 kg, 13% gained ≥3 kg and 11% gained ≥4 kg. Not surprisingly, these percentages were higher among subgroups with greater IDWGs. However, these weight gain estimates are conservative. The weight gain calculation cohort excluded patients who did not have 13 contiguous outpatient treatments in the studied period. Furthermore, we calculated weight gain over a single month. Such weight gains would snowball over time. These results indicate that fluid-related weight gain could be a potential unintended consequence of UF rate threshold implementation.

Our findings also underscore the importance of developing effective IDWG reduction programs. Strict dietary salt reduction has been demonstrated to lower IDWG and blood pressure [21, 22]. Dialysis facilities incorporate salt and fluid restriction counseling into their dietary education programs. However, patients report difficulty adhering to these restrictions [10], and IDWGs remain high [18]. Patient-acceptable, evidence-based education programs are needed. Beyond curbing dietary sodium intake, aligning the dialysate sodium with the serum sodium to avoid sodium-loading patients may be a viable approach to IDWG reduction [23]. However, close attention must be paid to potential hemodynamic consequences [24, 25]. Furthermore, our results emphasize the importance of revisiting the acceptance of shorter dialysis TTs by U.S. nephrologists. Several observational studies have suggested greater mortality risk from shorter TTs [20, 26, 27]. Experience from Australia and New Zealand, and other countries, demonstrates that longer TTs can be achieved under the in-center, thrice-weekly treatment paradigm [28]. In our opinion, implementation of longer TTs for appropriate patients is feasible in the current U.S. dialysis delivery system. Modest clinic operations modifications, staff education and improved patient counseling that begins well in advance of dialysis initiation may facilitate acceptance and implementation of longer TTs.

Most importantly, our findings highlight the critical importance of identifying and validating objective measures of volume status. Such tools will facilitate accurate estimation and prescription of target weight. Physical examination findings such as hypertension and edema have proven to be unreliable volume surrogates [29, 30]. Volume assessment tools such as bioimpedance, blood volume monitors and lung ultrasound lack validated clinical protocols and are not widely used. Once volume assessment tools are validated and incorporated into practice, target weight achievement will serve as an excellent measure of facility-level fluid management practices and an appropriate counter-balance for UF rate limitations. In our opinion, accurate volume assessment is a critical unmet need in dialysis care.

A target weight quality measure may help minimize untoward effects of UF rate reduction by prompting providers to: 1) focus on extracellular volume status, 2) more frequently re-assess target weight, 3) emphasize IDWG minimization and 4) administer extra or longer treatments when greater IDWGs occur. Thus, despite its limitations, the proposed target weight measure may provide an important check and balance for the UF rate measure. Measure detractors were concerned that the measure might be too easy to manipulate [7, 31]. For example, providers could adjust prescribed target weights rather than altering care to promote original target weight achievement. Consideration of the target weight measure as a QIP *reporting* measure might reduce this risk while also providing a means to track UF rate measure safety concerns. Second, future advances in transmission of clinical data to CMS might allow more granular, real-time tracking of clinical activity that would limit opportunities for gaming. Third advances in objective volume status measurement tools will likely facilitate incorporation of an extracellular volume measure into the QIP. In the meantime, the standardized hospitalization and readmission ratios may provide some protection against UF rate measure harms but are imperfect surrogates [32]. Practitioners must use clinical judgement when applying UF rate limitations, weighing the risks of overly rapid fluid removal with the risks of volume expansion on an individual patient-to-patient basis.

Strengths of our study include the large, nationally representative cohort with detailed clinical data and real-world application of proposed clinical quality measure criteria. Our study has limitations. First, we considered data from a single dialysis provider. Clinical protocols may differ across organizations, raising the possibility that individual and facility target weight patterns may vary across organizations. Related, target weights were prescribed by treating nephrologists, and we lacked data on how target weights were estimated and prescribed. Clinical approach to target weight estimation likely varied across providers. Second, we estimated the theoretical amount of fluid-related weight gain that would occur if a prescribed UF rate threshold of 13 mL/h/kg were implemented. In these calculations, we used observed IDWGs and prescribed TTs and assumed that these parameters would remain unchanged after UF rate threshold application. We were unable to account for possible IDWG reduction or TT extension that might occur in response to UF rate threshold implementation in a real-world clinical environment. Third, we lacked data on residual kidney function and anti-hypertensive medication use. Finally, we used target weight and UF rate definitions and selection criteria according to KCQA specifications, and results cannot be generalized to excluded patients or facilities.

## Conclusions

In conclusion, our data highlight the importance of reconsidering the target weight measure or developing a new measure that could serve as a counter-balance to the UF rate clinical performance measure. As we strive to protect patients from the consequences of overly aggressive fluid removal, we must apply equal vigilance to protecting them from the harms of under aggressive fluid removal. Pilot studies of the proposed fluid-related quality measures are needed.

## Additional files

Additional file 1: Table S1. Comprehensive description of the target weight measure. **Table S2.** Detailed description and representative example of the theoretical fluid-related weight gain accumulation calculations. **Table S3.** Patient and facility-level target weight achievement as calculated by target weight measure criteria on a monthly and annual basis. **Table S4.** Patient-level post-dialysis and target weight difference on a monthly basis stratified by above and below target weight categorization. **Figure S1.** Monthly mean facility target weight measure scores across 2012. (DOCX 1536 kb)

## Abbreviations

CMS: Centers for Medicare and Medicaid Services
IDWG: Interdialytic weight gain
KCQA: Kidney Care Quality Alliance
NQF: National Quality Forum
QIP: Quality Incentive Program
TT: Treatment time
U.S.: United States
UF: Ultrafiltration
