# Supplementary material for: Target weight achievement and ultrafiltration rate thresholds: potential patient implications
Source: BMC Nephrol. 2017 Jun 2;18:185. doi: 10.1186/s12882-017-0595-5 (PMC5457585; doi:10.1186/s12882-017-0595-5)
Supplement: Supplementary file 1 — Comprehensive description of the target weight measure. Table S2. Detailed description and representative example of the theoretical fluid-related weight gain accumulation calculations. Table S3. Patient and facility-level target weight achievement as calculated by target weight measure criteria on a monthly and annual basis. Table S4. Patient-level post-dialysis and target weight difference on a monthly basis stratified by above and below target weight categorization. Figure S1. Monthly mean facility target weight measure scores across 2012. (DOCX 1536 kb) [file 12882_2017_595_MOESM1_ESM.docx]

**Additional file**

**Table S1.** Comprehensive description of the target weight measure.^7^

| **Description** | % of patients in the facility with an average post-dialysis weight ≥1 kg above or below the prescribed target weight |
| --- | --- |
| **Time window** | 12 months |
| **Numerator** | # of patient-months in the facility with an average post-dialysis weight ≥1 kg above or below the prescribed target weight |
| **Numerator details** | For all patients meeting the denominator inclusion criteria in the reporting month:  1. Calculate the difference between the post-dialysis weight and the prescribed target weight for each treatment in the calculation period (during the week of the Kt/V assessment)  Difference_Treat_ = (post-dialysis weight (kg) – prescribed target weight (kg))  2. Take the sum of the differences calculated in step 1.  Difference_Sum_ = (Difference_Treat1_ + …… Difference_Treatj_)  j=Number of treatments during the week that the monthly Kt/V is drawn  3. Calculate each patient’s average weight difference for all HD treatments during the week that the monthly Kt/V is drawn:  Difference_Average_ = Difference_Sum_ / j  j=Number of treatments during the week that the monthly Kt/V is drawn  4. Sum the number of patients in each facility with an average dialysis treatment post-weight and target weight difference during the calculation period that is ≥ ±1 kg |
| **Denominator** | Total number of adult, in-center HD patients at the reporting facility meeting selection criteria |
| **Denominator details** | Identify all in-center HD patients who are ≥18 years in the facility during the reporting period |
| **Exclusions** | 1. PD or home HD patients  2. Age <18 years  3. Treatment without post-weight or target weight data  4. Patient present at reporting facility <30 days  5. Patient at facility with <11 patients during month  6. <7 HD treatments during reporting month |
| **Score type** | Rate / proportion |
| **Interpretation** | Lower score is more favorable |
| **Algorithm** | Scores are calculated on a monthly basis and then averaged over a 12-month period using the following algorithm to obtain the facility annual score:  1. Sum the number of patients in the denominator for each facility month  2. Sum the number of patients in the numerator for each facility month  3. Calculate the facility’s score for each month:    Score_k_ = Numerator Population_k_ / Denominator Population_k_  k=Each month of the reporting year  4. Calculate the facility’s annual score:  Annual Score = (Score_1_ + … Score_k_) / k  k=Number of months in the reporting year |

Abbreviations: HD, hemodialysis; PD, peritoneal dialysis.

**Table S2.** Detailed description of individual fluid-related weight accumulation with the application of a 13 mL/h/kg UF rate threshold.

|  | **Actual Data** | | | | | **Calculated Data after application of an UF rate threshold of 13 mL/h/kg** | | | |
| --- | --- | --- | --- | --- | --- | --- | --- | --- | --- |
| **HD tmt** | **Previous treatment post-dialysis weight (kg)** | **Pre-dialysis weight (kg)** | **IDWG (kg)** | **Prescribed TT (h)** | **Prescribed UF rate (mL/h/kg)** | **Maximum allowable UF (kg)** | **Weight change (kg)** | **Cumulative weight change (kg)** | **Calculated post-dialysis weight (kg)** |
| 1 | 62.7 | 64.4 | 1.7 | 4 | 6.72 | 3.26 | 0.00 | 0.00 | 62.70 |
| 2 | 60.2 | 65.5 | 5.3 | 4 | **21.83** | 3.26 | +2.04 | 2.04 | 64.74 |
| 3 | 62.7 | 66.4 | 3.7 | 4 | **14.64** | 3.37 | +0.33 | 2.37 | 65.07 |
| 4 | 62.4 | 64.8 | 2.4 | 4 | 9.54 | 3.38 | -0.98 | 1.39 | 64.09 |
| 5 | 60.7 | 64.8 | 4.1 | 4 | **16.75** | 3.33 | +0.77 | 2.16 | 64.86 |
| 6 | 61.0 | 66.4 | 5.4 | 4 | **21.95** | 3.37 | +2.03 | 4.18 | 66.88 |
| 7 | 63.3 | 66.3 | 3.0 | 4 | 11.76 | 3.48 | -0.48 | 3.71 | 66.41 |
| 8 | 61.9 | 65.4 | 3.5 | 4 | **14.02** | 3.45 | +0.05 | 3.75 | 66.45 |
| 9 | 61.7 | 65.3 | 3.6 | 4 | **14.47** | 3.46 | +0.14 | 3.90 | 66.60 |
| 10 | 60.8 | 63.1 | 2.3 | 4 | 9.38 | 3.46 | -1.16 | 2.73 | 65.43 |
| 11 | 60.1 | 63.7 | 3.6 | 4 | **14.85** | 3.40 | +0.20 | 2.93 | 65.63 |
| 12 | 61.1 | 65.5 | 4.4 | 4 | **17.86** | 3.41 | +0.99 | 3.92 | 66.62 |

In order to calculate the potential fluid-related weight gain based on a 13 mL/h/kg UF rate threshold, treatments were restricted to those occurring in February 2012. Patients were required to have no missing treatments during the month (n treatments= 12 or 13 depending on dialysis schedule) and were required to survive 60 days after the end of February 2012. The actual prescribed UF rate (mL/h/kg) for each treatment was calculated as follows: (pre-dialysis weight from current treatment – post-dialysis weight from previous treatment (mL)) / prescribed TT (h) / post-dialysis weight from previous treatment (kg). We established the maximum ultrafiltration volume (kg) allowable with application of a UF rate maximum of 13 mL/h/kg as follows: (13)(calculated post-weight from previous treatment)(prescribed TT) = maximum allowable UF (kg). The maximum allowable UF varied due to fluctuations in post-weights after application of calculated fluid-related weight gains and losses. The per treatment weight change (kg) was calculated as follows: actual IDWG (kg) – maximum allowable UF (kg). The cumulative fluid-related weight change (kg) was calculated by summing the weight changes (kg) of all previous treatments. The calculated post-dialysis weight (kg) was calculated by adding the weight change (kg) to the previous calculated post-dialysis weight. An example of these calculations applied to a single representative patient for all February 2012 treatments is shown in the table.

Abbreviations: HD, hemodialysis; tmt, treatment; IDWG, interdialytic weight gain; TT, treatment time; UF, ultrafiltration.

**Table S3.** Patient and facility-level target weight achievement as calculated by target weight measure criteria on a monthly and annual basis.^a^

|  |  | **Patient-level** | **Facility-level** |
| --- | --- | --- | --- |
| **Calculation period** | **N** | **Post-dialysis and target weight**  **difference (kg)** | **Percentage of facility patients**  **with average post-dialysis weight ≥1 kg above or below the prescribed target weight** |
| January | 107,951 | 0.3 ± 1.7;  0.2 [-0.3, 0.8] | 29.0 ± 11.9;  28.6 [20.7, 36.4] |
| February | 108,059 | 0.3 ± 1.7;  0.1 [-0.3, 0.8] | 28.2 ± 12.0;  27.5 [20.0, 35.8] |
| March | 110,058 | 0.3 ± 1.7;  0.1 [-0.3, 0.8] | 28.0 ± 11.7;  27.3 [19.6, 34.9] |
| April | 109,984 | 0.3 ± 1.6;  0.1 [-0.3, 0.8] | 27.2 ± 11.6;  26.3 [18.9, 34.5] |
| May | 112,037 | 0.3 ± 1.6;  0.1 [-0.3, 0.7] | 26.6 ± 11.5;  25.5 [18.4, 33.8] |
| June | 112,670 | 0.2 ± 1.6;  0.1 [-0.3, 0.7] | 26.3 ± 11.2;  25.7 [18.8, 33.3] |
| July | 113,449 | 0.2 ± 1.6;  0.1 [-0.3, 0.7] | 26.1 ± 11.0;  25.5 [18.2, 33.3] |
| August | 115,081 | 0.2 ± 1.6;  0.1 [-0.3, 0.7] | 25.7 ± 11.0;  25.0 [18.1, 32.5] |
| September | 114,504 | 0.3 ± 1.6;  0.1 [-0.3, 0.7] | 26.3 ± 11.3;  25.6 [18.3, 33.3] |
| October | 115,503 | 0.3 ± 1.6;  0.1 [-0.3, 0.7] | 26.7 ± 11.0;  26.0 [18.8, 33.3] |
| November | 115,587 | 0.3 ± 1.6;  0.1 [-0.3, 0.8] | 27.3 ± 11.7;  26.9 [19.2, 34.6] |
| December | 116,056 | 0.3 ± 1.6;  0.1 [-0.3, 0.8] | 26.9 ± 11.5;  26.3 [18.8, 34.0] |
| Annual | 152,196 | 0.3 ± 1.2;  0.1 [-0.2, 0.6] | 27.1 ± 9.7;  26.4 [20.3, 32.9] |

^a^ Values presented as means ± SDs; medians [quartile 1, quartile 3].

**Table S4.** Patient-level post-dialysis and target weight difference on a monthly basis stratified by above and below target weight categorization.^a^

|  | **≥1 kg *below* target weight** | | **≥1 kg *above* target weight** | |
| --- | --- | --- | --- | --- |
|  | **n** | **Post-dialysis and target weight difference (kg)** | **n** | **Post-dialysis and target weight difference (kg)** |
| January | 9,451 | -2.5 ± 2.1;  -1.8 [-2.8, -1.3] | 22,800 | 2.4 ± 1.8;  1.9 [1.3, 2.8] |
| February | 9,276 | -2.4 ± 1.9;  -1.8 [-2.8, -1.3] | 22,066 | 2.4 ± 1.8;  1.8 [1.3, 2.8] |
| March | 9,655 | -2.5 ± 2.0;  -1.8 [-2.9, -1.3] | 21,943 | 2.4 ± 1.8;  1.8 [1.3, 2.8] |
| April | 9,681 | -2.5 ± 2.0;  -1.8 [-2.8, -1.3] | 21,021 | 2.4 ± 1.8;  1.8 [1.3, 2.7] |
| May | 9,951 | -2.4 ± 1.9;  -1.8 [-2.8, -1.3] | 20,678 | 2.4 ± 1.7;  1.8 [1.3, 2.8] |
| June | 10,298 | -2.4 ± 1.9;  -1.7 [-2.7, -1.3] | 20,160 | 2.4 ± 1.8;  1.8 [1.3, 2.7] |
| July | 10,638 | -2.4 ± 1.8;  -1.7 [-2.7, -1.3] | 19,812 | 2.4 ± 1.7;  1.8 [1.3, 2.7] |
| August | 10,226 | -2.3 ± 1.8;  -1.7 [-2.7, -1.3] | 20,231 | 2.4 ± 1.8;  1.8 [1.3, 2.7] |
| September | 9,778 | -2.4 ± 1.9;  -1.7 [-2.7, -1.3] | 21,131 | 2.4 ± 1.8;  1.8 [1.3, 2.8] |
| October | 9,466 | -2.4 ± 1.8;  -1.8 [-2.8, -1.3] | 22,118 | 2.4 ± 1.8;  1.8 [1.3, 2.7] |
| November | 9,517 | -2.4 ± 1.8;  -1.7 [-2.7, -1.3] | 22,971 | 2.4 ± 1.8;  1.8 [1.3, 2.8] |
| December | 9,544 | -2.4 ± 1.8;  -1.7 [-2.7, -1.3] | 22,368 | 2.4 ± 1.8;  1.8 [1.3, 2.7] |

^a^ Values presented as means ± SDs; medians [quartile 1, quartile 3].

**Figure S1.** The monthly mean facility target weight measure scores across 2012.


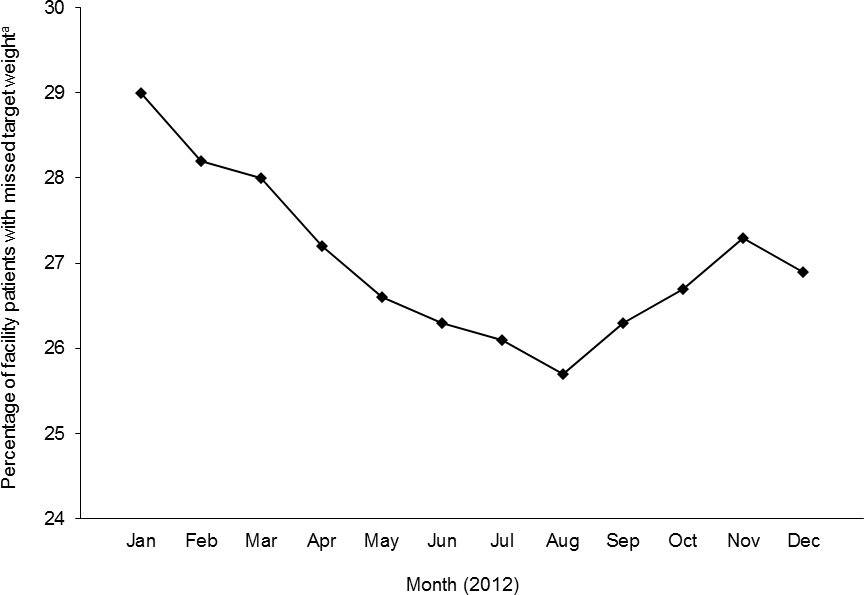


^a^ Missed target weight defined as average post-dialysis weight ≥1 kg above or below prescribed target weight in the calculation period (week of the monthly Kt/V assessment).
